# From Technical Performance to Assistive Validity: A Critical Review of Evaluation Approaches for AI-Enabled Assistive Technology

**DOI:** 10.3390/healthcare14182900

**Published:** 2026-09-08

**Authors:** Mabel Giraldo, Fabio Sacchi

**Affiliations:** Department of Human and Social Sciences, University of Bergamo, 24129 Bergamo, Italy; fabio.sacchi@unibg.it

**Keywords:** assistive technology, artificial intelligence, AI-enabled assistive technology, evaluation approach, outcome assessment, person-centred assessment, participation, agency, assistive validity

## Abstract

**Background/Objectives**: The increasing integration of artificial intelligence (AI) into assistive technologies challenges evaluation models traditionally centred on usability, satisfaction and device-related outcomes. Because *AI-enabled systems* are probabilistic, data-dependent and adaptive, their evaluation must also address algorithmic behaviour, user agency and consequences in everyday life. This critical review aimed to identify and compare standardised instruments, frameworks and structured procedures for evaluating *AI-enabled assistive technologies* and to determine the extent to which they connect AI-specific properties with the goals of persons with disabilities, activities, participation and environmental conditions. **Methods**: MEDLINE/PubMed, Scopus and IEEE Xplore were searched without publication-date restrictions, with final searches completed on 30 June 2026 and complemented by backward and forward citation searching. Data were extracted on approach type, purpose, stage of application, target populations and technologies, methodological evidence, evaluated dimensions and outcomes. Evidence was classified as documented, partially documented, not documented or not applicable, while dimensional coverage was coded as explicit, partial or absent. **Results**: Of the 2299 records screened by title and abstract, 478 publications underwent full-text assessment and 45 were retained in the documentary corpus. These publications supported 16 evaluation approaches: four frameworks, three structured procedures and nine measurement instruments published between 2001 and 2026. The person-related dimension was explicitly operationalised in 15 approaches and activity in nine, whereas participation was explicit in only one and environmental factors in six. AI-specific properties were explicitly evaluated in three approaches, and everyday-life outcomes over time in two. Among the approaches included in this review, none combined comprehensive coverage of person, activity, participation and environment with AI-specific evaluation and longitudinal monitoring. **Conclusions**: Evaluation remains fragmented across assistive technology, human–computer interaction, human–robot interaction and AI assessment traditions. The review proposes assistive validity as a higher-order criterion linking technical performance, agency and meaningful outcomes for persons with disabilities. It also identifies a modular and longitudinal evaluation architecture as a priority for future empirical development and validation.

## 1. Introduction

Assistive technologies encompass products, systems and services intended to maintain or improve the functioning, independence and participation of persons with disabilities [1,2]. Their value lies in the interaction between the characteristics of technology, the person, the activities pursued and the context of use, rather than in the product considered in isolation; what constitutes a positive outcome depends on each person’s goals, preferences and experience [3]. Maximising the contribution of these technologies to participation therefore requires a person-centred approach that begins with the person’s goals and the activities that are meaningful to them, and situates the clinical, functional and technical dimensions within a process oriented towards everyday life [4,5].

This perspective is particularly relevant today, given the increasing integration of artificial intelligence into assistive technologies. This integration has taken the form of a gradual process, without a single founding moment, that has affected the various families of assistive technologies in different ways: an initial phase relied primarily on rule-based systems, statistical models and sensor- and automated-control solutions, while subsequent developments drew on machine learning, deep learning and, more recently, generative models [6]. The term *AI-enabled assistive technologies* refers to assistive technologies whose functioning depends, at least in part, on systems capable of classifying information, recognising patterns, making predictions, generating content or adapting their behaviour to the person and the conditions of use [7,8].

AI expands the range of available functions and changes how technology mediates the relationship between the person and the activity, as the system may interpret data, anticipate needs, generate messages, or intervene in the selection and execution of an action [9,10]. This transformation introduces issues that extend beyond interface usability. Outputs are probabilistic and may appear plausible even when inaccurate [11]; performance depends on the representativeness of the data and may deteriorate when particular characteristics are poorly represented or under adverse conditions of use [12,13]. AI also affects the control exercised by the person, raising questions concerning choice, agency, privacy and dependence on the system in activities closely connected to identity and self-determination [14,15]. Hence, high technical performance leaves unresolved the issue of whether the system is understandable, controllable and consistent with individual priorities [16].

The evaluation of assistive technologies must therefore establish for whom, in relation to which goals, in which activities and with what consequences a technology produces a benefit. The conceptual models used in the field provide a framework for addressing this question. The *International Classification of Functioning* interprets functioning as the outcome of the interaction between the person, activities, participation and contextual factors [17]. The *outcome model developed* by Fuhrer [18], the *Matching Person and Technology model* [19] and the *Human Activity Assistive Technology model* [20] likewise conceptualise assistive outcomes in relation to the fit between the person, the technology, the activities undertaken and the environment in which technology use occurs. The literature on operational tools nevertheless remains broad and heterogeneous: Tao [21] identified 159 instruments covering 103 constructs, while Borgnis [22] identified 53 outcome measures, revealing uneven coverage of relevant dimensions and limited comparability across studies. This heterogeneity is compounded by fragmentation across distinct traditions—assistive-technology assessment, human–computer interaction, human–robot interaction and frameworks addressing the properties of AI systems—which illuminate separate aspects of the phenomenon.

*AI-enabled assistive technologies* lie precisely at the intersection of these fields, and their evaluation requires system behaviour, the person’s experience and the assistive value produced to be considered jointly. Reviews conducted to date have mainly examined general instruments, outcome measures, or individual application domains, and it remains unclear to what extent the available approaches connect the properties of AI with a person’s characteristics, goals, activities, participation, and environmental factors. The present review aims to identify and analyse standardised instruments, frameworks and structured procedures for evaluating AI-enabled assistive technologies identified through the adopted search and source-selection strategy, examining their methodological characteristics, purposes, application stages, operationalised dimensions and assessed outcomes.

The review is guided by the following question: which evaluation approaches for *AI-enabled assistive technologies* are identified through the adopted review strategy, and to what extent do they connect AI-specific properties with the person’s goals, activities, participation and environmental factors?

Three sub-questions follow. (1) What methodological characteristics, target populations and technologies, purposes and stages of application characterise the identified evaluation approaches? (2) To what extent do these approaches operationalise the specific properties of artificial intelligence and the dimensions relating to the person, activity, participation and environmental factors, and how do they represent the interactions among them? (3) Which outcomes are assessed, and to what extent do the identified approaches document changes associated with technology use in everyday-life contexts and over time, with particular reference to the person’s goals, activities and participation?

## 2. Methods

### 2.1. Review Design and Scope

A critical review design was selected because the field is methodologically heterogeneous and spans several disciplinary traditions. Critical reviews combine the structured identification and examination of relevant literature with an interpretive analysis of its theoretical assumptions, methodological orientations, strengths and limitations, rather than limiting the synthesis to a descriptive inventory of available evidence [23]. This design was therefore appropriate for comparing evaluation approaches developed within assistive-technology assessment, human–computer interaction, human–robot interaction and AI evaluation, whose purposes, units of analysis and forms of evidence are not directly equivalent. The review aimed to connect these approaches within an overarching interpretive account and to identify recurring conceptual and methodological disconnections, an orientation consistent with conceptual synthesis and theory building in emerging and fragmented fields [24,25].

The process comprised four integrated phases [25]: (a) definition of scope and selection criteria; (b) search and source selection; (c) data extraction and critical appraisal; and (d) comparison and interpretive synthesis. The units of analysis were the evaluation approaches identified in the literature, classified as measurement instruments, frameworks or structured procedures. For each approach, the primary publication describing its development was examined, along with validation or application studies when available and relevant to the review questions.

No protocol for this critical review was prospectively registered. The review questions, eligibility criteria and analytical coding rules reported in Section 2 document the methodological decisions used for source selection and comparative analysis, while higher-order interpretive categories were developed during the synthesis as described in Section 2.4.

### 2.2. Literature Search and Source Selection

The literature search was developed and progressively refined between January and June 2026. The final searches were conducted in MEDLINE/PubMed, Scopus and IEEE Xplore on 30 June 2026, with no restrictions on publication date. These databases were selected to provide multidisciplinary coverage across biomedical and rehabilitation research, engineering and computer science, including literature relevant to assistive technology, human–computer interaction, human–robot interaction and *AI-enabled systems*. The database search was complemented by backward and forward citation searching and manual reference-list inspection to broaden source identification across these intersecting fields. The strategy combined four groups of terms, adapted to each database:

(“assistive technolog*” OR “assistive device*” OR “assistive system*” OR “assistive robot*” OR “socially assistive robot*” OR “AI-enabled assistive technolog*” OR “accessibility technolog*”) AND (“artificial intelligence” OR “machine learning” OR “deep learning” OR “computer vision” OR “natural language processing” OR “large language model*” OR “generative AI” OR “intelligent system*” OR “adaptive system*”) AND (“evaluation instrument*” OR “measurement instrument*” OR scale* OR questionnaire* OR framework* OR protocol* OR checklist* OR evaluat* OR assess* OR outcome* OR usabil* OR accept* OR trust OR explainab* OR safety) AND (disab* OR impair* OR rehabilitation OR “older adult*” OR dementia OR blind* OR deaf* OR aphasia OR “augmentative and alternative communication”).

The complete database-specific search strategies, including the fields searched, syntax adaptations, search dates, and the number of records retrieved, are reported in Supplementary Table S1.

The database search was complemented by backward and forward citation searches and manual inspection of reference lists to identify publications describing the development, validation, or application of potentially eligible evaluation approaches.

English-language publications were included when they described evaluation approaches relevant to *AI-enabled assistive technologies* and sufficiently formalised for comparative analysis. Specifically, the review considered (a) instruments, frameworks or procedures developed for *AI-enabled assistive technologies*, AI accessibility or assistive robotics; (b) approaches originally developed in assistive technology, provided at least one application to an AI-based system was documented; and (c) instruments from human–computer or human–robot interaction applied to an intelligent assistive technology or incorporated into an included framework. A technology was considered “AI-enabled” when its assistive function depended on perception, classification, prediction, generation, planning, adaptation, or automated action selection; approaches evaluating such functions prospectively through Wizard-of-Oz simulations were included when the simulation supported the development or testing of a future automated component. Approaches also had to describe their domains, items, judgement criteria or operational stages in sufficient detail. The review excluded ad hoc questionnaires not reported in full, isolated technical metrics, recommendations without operational criteria, general AI frameworks that did not address accessibility or assistive technologies, and diagnostic instruments. Literature reviews and application studies were used to identify and contextualise approaches, without being treated as independent units of analysis. After duplicate removal, title and abstract screening and subsequent full-text assessment were conducted independently by the two researchers using the predefined eligibility criteria. Differences in eligibility judgments were discussed until consensus was reached.

Screening was conducted at the publication level, whereas inclusion in the comparative synthesis was determined at the level of the evaluation approach. Publications describing the development, validation or application of the same approach were therefore linked to a single unit of analysis. Backward and forward citation searching and manual reference-list inspection were used to identify additional publications relevant to potentially eligible approaches and to retrieve supporting development, validation or application studies. The selection process was documented at both screening stages. Of the 2299 records undergoing title and abstract screening, 478 publications proceeded to full-text assessment. Following full-text assessment, 45 publications were retained as the documentary corpus from which the eligible evaluation approaches and their supporting development, validation or application evidence were identified. Because the review was organised analytically at the level of the evaluation approach rather than the individual publication, these publications were subsequently linked to the corresponding approaches during source chaining and evidence profiling.

### 2.3. Data Extraction and Critical Appraisal

For each included approach, publications on development, validation and subsequent applications were retrieved where available, and information was extracted using a form derived from the research questions. The form recorded (1) identifying information (name, authors, place and year, type, field of use, purpose, validation/application); (2) methodological characteristics, including participants, administration and evidence of validity, reliability, responsiveness, cross-cultural adaptation and feasibility; (3) target population and technology, with attention to the AI component; (4) dimensions relating to the person, environmental factors and the ICF domains of activity and participation; and (5) outcomes concerning the person’s experience, technology use and integration into everyday life. Information on choosing and controlling how the technology operated, understanding automated functions, intervening in the output and discontinuing use was extracted within personal factors and the user experience, and later drawn together under agency as a cross-cutting category.

Data extraction was conducted by R1 using the predefined form and independently verified by R2. Differences concerning the extracted information or its classification were discussed by the two researchers and resolved by consensus.

The availability of evidence was classified, separately for each property, as documented (an empirical evaluation directly addressing the property, with methods and results), partially documented (indirect evidence, restricted samples or components, or evidence derived mainly from literature, expert judgement or stakeholder involvement), not documented, or not applicable. Criteria were adapted to the type of approach: standardised and individualised instruments were appraised on their psychometric properties (validity, internal consistency, test–retest stability, inter-rater agreement, responsiveness, cross-cultural adaptation, feasibility), with repeated administration for stability or use in pre–post studies counted as only partial evidence of responsiveness; frameworks and structured procedures, generally lacking items or scores, were appraised instead on content relevance and coherence, transparency of development, clarity of domains and stages, whole-approach empirical application, reproducibility across evaluators or contexts, and feasibility. For frameworks and procedures, evidence relating to incorporated instruments was not transferred to the approach as a whole. The appraisal examined the consistency and adequacy of the available evidence—degree of formalisation, clarity of constructs, transferability, feasibility and ability to capture real-world change—rather than the methodological quality of individual studies.

To improve the auditability of these classifications, the decision rules used to assign D, PD, ND and NA codes are reported in Supplementary Note S1. The rules specify the evidentiary threshold for documented and partially documented evidence, the treatment of secondary validation or application studies, and the handling of evidence that differed across publications relating to the same evaluation approach. Where findings differed across sources, the classification reflected the overall documentary profile rather than transferring the most favourable result from a single publication to the approach as a whole.

### 2.4. Comparative Analysis and Interpretive Synthesis

The extracted information was organised into comparative matrices and coded deductively. For each approach, coverage of the dimensions relating to the person, activity and participation, AI-specific properties and the environment was classified as explicit (E), when the dimension formed an independent domain, item set or procedural stage; partial (P), when addressed through individual items, indirect indicators or proxies; or absent (A).

For the person-related dimension, the coding also considered how the intended technology user’s perspective was represented. Coverage was classified as explicit when personal characteristics, goals, experiences, or responses were directly elicited from the intended users or independently operationalised within the approach; it was classified as partial when the person’s perspective was represented mainly through proxy respondents, simulations, expert judgement or inferred user characteristics.

Interaction among components was classified as explicit when a formally described mechanism connected the technology with one or more other components—person, activity or task, participation, or environment—through matching, structured scenarios, task-based trials, testing sequences or relational models; partial when the relationship could be inferred only from overall judgements concerning the product, its functions or its effects; and absent when the components were assessed separately or no relationship among them was described. An E code indicated the presence of a formalised connection rather than comprehensive coverage of all the dimensions considered. All E/P/A coding was performed using the predefined criteria and independently verified by both researchers. To quantify the reproducibility of the dimensional coding, the 16 included approaches were independently coded by the two researchers across the seven comparative dimensions before consensus resolution. Inter-rater agreement was assessed using Cohen’s kappa and showed substantial agreement (κ = 0.81). Differences in coding were subsequently discussed and resolved by *consensus*.

The analysis also recorded the evaluation stage addressed by each approach: needs assessment and selection, technical verification, evaluation of everyday use, and outcome monitoring over time. A cross-cutting interpretive synthesis was then conducted by comparing the patterns identified in the matrices across disciplinary traditions. The comparative dimensions relating to person, activity, participation, environment, interaction among components, AI-specific properties and everyday-life outcomes over time were defined deductively before the interpretive synthesis and used to organise the descriptive comparison of the included approaches. Higher-order concepts such as agency, dynamic matching and assistive validity were not additional a priori coding dimensions. They were developed during the interpretive synthesis by examining recurring relationships and disconnections across the predefined dimensions and by relating these patterns to established assistive technology and validity concepts. Accordingly, assistive validity is presented as an interpretive proposal arising from the comparative synthesis rather than as a construct independently demonstrated by the coding framework itself.

Particular attention was given to recurring disconnections between (a) person-centred assessment and the evaluation of algorithmic behaviour; (b) acceptance or satisfaction and the person’s ability to understand, control or interrupt automated functions; (c) activity-level evaluation and participation or environmental outcomes; and (d) immediate interaction outcomes and changes sustained in everyday life over time. These patterns were subsequently interpreted in relation to established models of assistive technology, functioning, agency and outcome evaluation. This process informed the higher-order categories developed in Section 4, including dynamic matching, agency and the relationship between technical performance and assistive outcomes.

## 3. Results

### 3.1. Selection Phase

Database searches conducted in MEDLINE/PubMed, Scopus and IEEE Xplore, without publication-date restrictions, yielded 2433 records. After removal of 128 duplicates and six non-English-language records, 2299 records underwent title and abstract screening. At this stage, 1821 records were excluded and 478 publications proceeded to full-text assessment. Of these, 433 were excluded because they did not meet one or more eligibility criteria, principally because the evaluation approach was not sufficiently formalised, the technology did not meet the operational definition of AI-enabled assistive technology, or no documented application consistent with the scope of the review was identified. The remaining 45 publications constituted the documentary corpus used to identify and characterise the eligible evaluation approaches and to retrieve relevant development, validation or application evidence through backward and forward citation searching and manual reference-list inspection.

**Figure 1:**
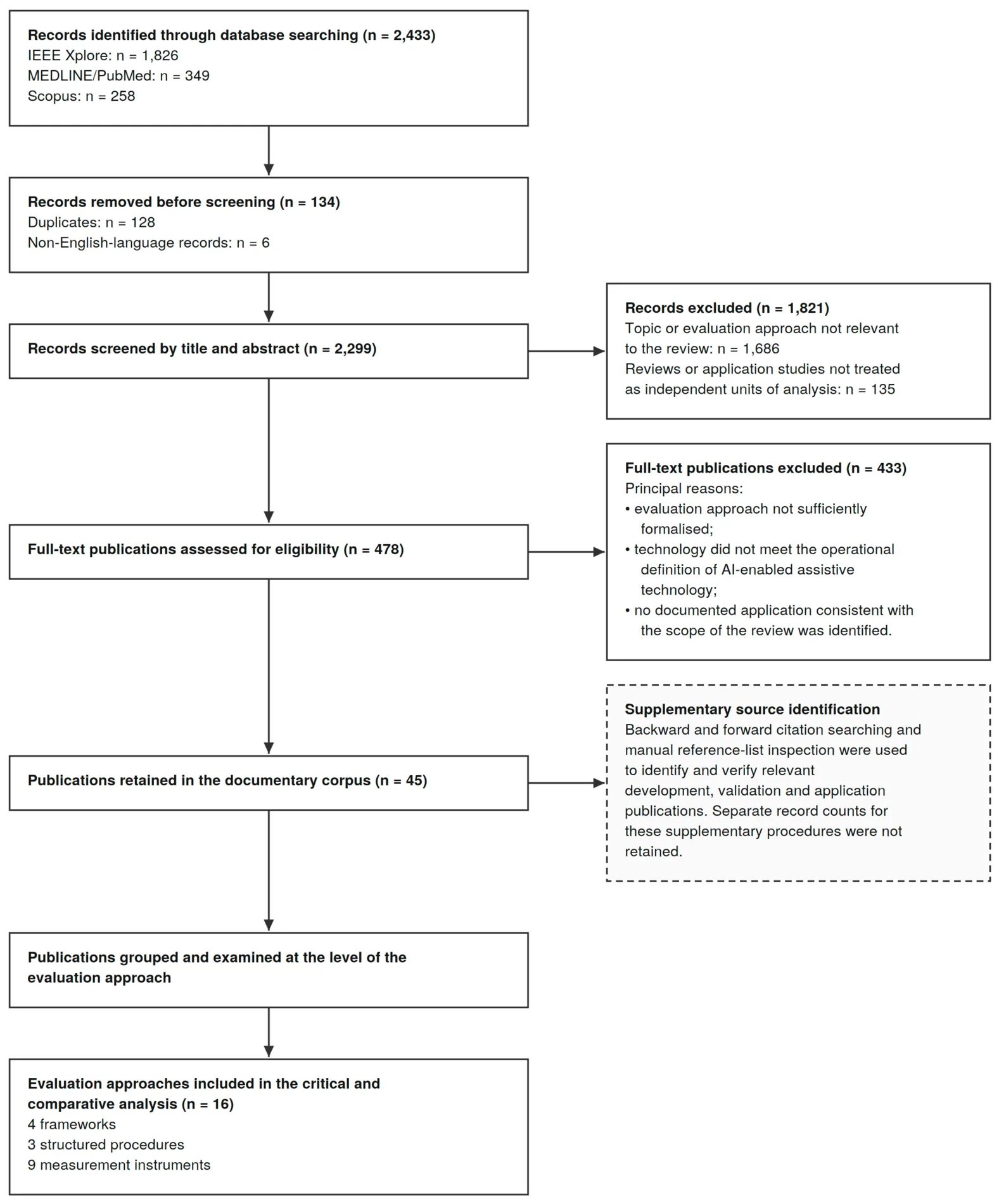
Selection and analytical workflow of the critical review.

The selection and analytical workflow is summarised in Figure 1.

Following source selection and citation chaining, 16 distinct evaluation approaches met the eligibility and operationalisation criteria and constituted the units of critical and comparative analysis. Screening and source identification were conducted at the publication level, whereas the final synthesis was organised at the level of the evaluation approach. Accordingly, multiple publications describing the development, validation or application of the same approach were linked to a single analytical unit. For each approach, the primary reference and, where available, selected validation or application publications were examined.

The number and type of publications supporting each evaluation approach varied according to its stage of development and subsequent use. Table 1 identifies the primary reference used to define and characterise each approach and, where available, the principal validation or application publications used in the comparative analysis. These supporting publications constitute the documentary basis for the evidence profiles reported in the review, but they are not treated as separate units of analysis. Table 1 is intended to make the relationship between publications and evaluation approaches transparent rather than to provide an exhaustive bibliography of every subsequent application of each approach.

The review identified 16 distinct evaluation approaches: four frameworks (the *AI-enabled AT Framework*, the *AI TEVV for Accessibility Framework*, the *AUSUS Evaluation Framework* and *SHARA-WoZ*), three structured procedures (*X-HAZOP*, *Value Preference Profiles and Ethical Compliance Quantification*, and the *Acceptance Test for Assistive Robots*) and nine measurement instruments. The latter comprise standardised self-report scales and questionnaires, an individualised interview, a structured assessment instrument and the questionnaire operationalising the *Almere acceptance model*. The approaches originate from assistive-technology assessment, outcome evaluation, human–robot interaction and technology-acceptance research. The primary reference for each approach, together with relevant validation or application publications where available, is reported. These publications provided the documentary basis for the analysis, while the evaluation approaches themselves constituted the units of comparison.

The approaches were introduced between 2001 and 2026. The review includes established instruments developed primarily within assistive-technology assessment and outcome evaluation, measures subsequently developed within technology acceptance and human–robot interaction research, and more recent frameworks and procedures addressing AI-related properties, risks, and modes of operation. This temporal distribution reflects the approaches identified through the eligibility criteria rather than constituting a comprehensive historical reconstruction of the field.

The included approaches also differed in their relationship to artificial intelligence and automation. For interpretive purposes, three broad groups can be distinguished. Five approaches were developed specifically around AI-enabled or automated assistive systems and their evaluation: the AI TEVV for Accessibility Framework, the AI-enabled AT Framework, Value Preference Profiles and Ethical Compliance Quantification, X-HAZOP and SHARA-WoZ, the latter using simulated levels of autonomy rather than requiring an implemented AI model. Seven approaches originated primarily within assistive robotics, human–robot interaction or intelligent-agent evaluation and assessed the technology largely at the level of the complete system: the Robot Era Inventory, AUSUS Evaluation Framework, UNRAQ, Acceptance Test for Assistive Robots, QUEAD, PYTHEIA and the Almere Model. Four established assistive-technology instruments—IPPA, PIADS, QUEST 2.0 and ATD PA—were technology-agnostic in their original formulation and were included because subsequent robotic or AI-enabled applications met the eligibility criteria. This stratification distinguishes the historical origin and intended object of evaluation of the approaches and prevents the absence of AI-specific properties in legacy instruments from being interpreted as a deficiency relative to purposes for which they were not originally designed.

The approaches address different and only partially overlapping stages of evaluation. *UNRAQ*, *ATD PA* and *IPPA* support the identification of needs, priorities or matching requirements before technology provision, although *ATD PA* and *IPPA* also allow subsequent reassessment. The *AI-enabled AT Framework* extends across design, selection, implementation and review, whereas the *AI TEVV for Accessibility Framework* addresses testing and monitoring throughout the system life cycle. AUSUS combines design, experimentation and field evaluation, while *X-HAZOP, Value Preference Profiles and Ethical Compliance Quantification*, and *SHARA-WoZ* are primarily intended for prospective analysis, prototyping or comparison of alternative system behaviours and levels of autonomy.

Other approaches are applied mainly during or after interaction with the technology. *PYTHEIA*, *QUEAD*, the *Robot Era Inventory* and the *Acceptance Test for Assistive Robots* focus predominantly on immediate experience or task performance, whereas QUEST 2.0 and *PIADS* may be administered following provision and at follow-up. The *Almere Model* can be used before interaction, immediately afterwards or following a period of use. Taken together, the approaches cover multiple stages of the evaluation process, but no single approach provides an integrated pathway from initial assessment and technical verification to longitudinal monitoring of everyday-life outcomes.

### 3.2. Extraction Phase

The methodological characteristics and evidence profiles of the included evaluation approaches are reported in Supplementary Table S2, organised into two panels by approach type. Panel A presents the standardised and individualised measurement instruments and reports evidence concerning validity, reliability, responsiveness, cross-cultural adaptation and feasibility. Panel B presents the frameworks, structured procedures, and reports evidence on development transparency and content relevance, the empirical application of the complete approach, procedural reproducibility, and feasibility. Evidence was classified as documented, partially documented, not documented or not applicable according to the criteria defined in Section 2.3.

These classifications describe the availability and degree of methodological documentation identified for each evaluation approach and should not be interpreted as a hierarchy of evidence strength or as a risk-of-bias assessment of the individual supporting studies.

The appraisal criteria were differentiated according to the type of evaluation approach. Measurement instruments were examined with respect to validity, reliability, responsiveness, cross-cultural adaptation, and feasibility. Frameworks and structured procedures were examined separately in relation to the transparency of their development, the relevance and coherence of their domains, the clarity of their operational stages, empirical application of the complete approach, procedural reproducibility and feasibility. Psychometric properties that were not applicable to frameworks or procedures were not treated as equivalent to these methodological characteristics. Stakeholder, technology-user, or expert involvement was considered evidence of content relevance, but not of empirical validation of the complete framework or procedure.

Among the measurement instruments, evidence of reliability was documented for *QUEST 2.0*, *ATD PA*, *PIADS*, the *Almere Model*, and *UNRAQ*, and partially documented for PYTHEIA, QUEAD, and the *Robot Era Inventory*. Consistent evidence of reliability was not identified for *IPPA*. For frameworks and structured procedures, psychometric reliability was considered not applicable; procedural reproducibility was therefore examined separately. Evidence of procedural reproducibility was generally not documented, with partial evidence identified only for *Value Preference Profiles and Ethical Compliance Quantification* regarding coding procedures and protocol verification.

Evidence addressing validity was documented for *QUEST 2.0*, *ATD PA*, *PIADS*, *IPPA* and the *Almere Model*, and partially documented for *PYTHEIA*, *QUEAD*, *UNRAQ* and the *Robot Era Inventory*. Frameworks and structured procedures were not classified according to psychometric validity. Their appraisal considered development transparency, content relevance and empirical application of the complete approach. Content relevance was most clearly supported for the *AI-enabled AT Framework*, the *AI TEVV for Accessibility Framework* and *Value Preference Profiles* and *Ethical Compliance Quantification*, whereas empirical application of the complete approaches was absent for the *AI-enabled AT Framework* and the *AI TEVV for Accessibility Framework*; preliminary, laboratory-based, simulation-based, proof-of-concept or brief controlled applications were available for the other recent approaches. For AUSUS, application extended beyond the initial *CLARC evaluation* to a subsequent real-world study, although evidence remained limited to a small number of systems and settings.

Among the measurement instruments, responsiveness was documented for IPPA and partially documented for PIADS and the *Almere Model*. No specific evidence of responsiveness was identified for *QUEST 2.0*, *ATD PA*, *PYTHEIA*, *QUEAD* or the *Robot Era Inventory*. *Responsiveness* was considered not applicable to *UNRAQ* because the questionnaire is primarily intended for preliminary assessment of needs, requirements and expectations rather than for measuring change. For frameworks and structured procedures, responsiveness was not treated as an applicable psychometric property; their capacity to support repeated or longitudinal evaluation was examined instead in the comparative analysis.

Formally documented cross-cultural adaptations were identified for *QUEST 2.0*, *ATD PA*, *PIADS* and the *Almere Model*. *IPPA* has been used in several countries, but consistent information on formal cross-cultural adaptation procedures was not identified; international or cross-national application alone was not treated as evidence of cross-cultural adaptation. Similarly, psychometric testing of the modified *N-UNRAQ* in a different national context was not coded as formal cross-cultural adaptation of the original *UNRAQ* because it represents a derivative version rather than a direct adaptation. Cross-cultural adaptation was not used as an appraisal criterion for frameworks and structured procedures, which were assessed according to methodological criteria appropriate to their format.

Feasibility was documented for the established measurement instruments used in clinical, rehabilitation or care settings, including *QUEST 2.0*, *ATD PA*, *PIADS*, *IPPA*, the *Almere Model* and *UNRAQ*. For *PYTHEIA*, *QUEAD* and the *Robot Era Inventory*, feasibility was partially documented because the available applications were mainly limited to small samples or controlled testing conditions. Among the structured procedures, the *Acceptance Test for Assistive Robots* demonstrated feasibility in a brief controlled trial. Feasibility was partially documented for *SHARA-WoZ*, *X-HAZOP*, *Value Preference Profiles* and *Ethical Compliance Quantification*, and the *AUSUS Evaluation Framework* because their applications were limited to laboratory studies, workshops, simulations, proof-of-concept evaluations or a limited number of systems and settings. Feasibility of the complete *AI-enabled AT Framework* and *AI TEVV for Accessibility Framework* has not yet been documented.

The target populations and technologies addressed by the included evaluation approaches are reported in Supplementary Table S3. For instruments originally developed within the broader field of assistive technology, the table also identifies the documented applications to *AI-enabled systems* that supported their inclusion in the review. The target population refers to the persons for whom the approach or technology was intended and does not necessarily correspond to the participants involved in its development or validation. The analysis distinguished among the general properties of the technology, the AI components incorporated into the system and the AI-specific properties evaluated. The distribution of target populations shows that the nine evaluation approaches are not restricted to a specific diagnostic condition and are intended for persons with disabilities, users of assistive technologies or groups defined in relation to the product under consideration. Five approaches target older adults or persons involved in services supporting older adults, while two specifically concern persons with dementia.

The technologies considered are predominantly robotic systems and include socially assistive robots, robotic rollators, manipulator arms and mobile platforms designed to support everyday activities. The *corpus* also includes wearable computer-vision devices, environmental monitoring and assistance systems and, within the cross-cutting frameworks, speech-, language-, vision-, prediction- and adaptation-based applications. The assistive functions addressed include mobility and manipulation, access to information, communication, monitoring, safety, cognitive stimulation, companionship and support for learning or carrying out everyday activities.

The general technology properties reported most frequently concern ease of use, usefulness, effectiveness or performance, safety, comfort, accessibility, perceived reliability and quality of interaction. In instruments originating in assistive-technology assessment, these properties are assessed mainly through satisfaction, matching or the overall effects attributed to the product. Approaches developed in robotics also consider control modes, individual robot functions, quality of interaction and behaviour observed during task performance. More recently developed frameworks additionally include dimensions relating to privacy, cybersecurity, equity, value, rights, ethical consequences and the allocation of control.

The artificial intelligence components incorporated into the technologies include computer vision, action recognition, language processing and generation, speech recognition and synthesis, autonomous navigation, environmental perception, planning, decision-making systems and adaptive functions. These components, however, are not always evaluated as distinct objects. AI-specific properties are explicitly operationalised in the *AI-enabled AT Framework*, the *AI TEVV for Accessibility Framework* and *Value Preference Profiles* and *Ethical Compliance Quantification*. They are considered partially in *X-HAZOP* and *SHARA-WoZ* respectively through the analysis of risks associated with autonomy and the comparison of different modes of operation. In the other eleven approaches, the algorithmic component is present in the technology or in the documented applications but is not evaluated separately from the overall characteristics of the product and the interaction.

Accuracy, robustness, bias, failure modes and variation in performance across groups and contexts are translated into specific testing stages and procedures only in the *AI TEVV for Accessibility Framework*. The *AI-enabled AT Framework* addresses quality, reliability, safety, equity, transparency and control at the level of principles. *Value Preference Profiles* and *Ethical Compliance Quantification* instead examines the alignment between automated actions and the person’s value preferences. The other instruments and approaches originating in assistive-technology assessment and human–robot interaction primarily assess the perceived or observable consequences of the system without separately identifying the contribution of the algorithmic component.

### 3.3. Comparative Analysis

The comparative analysis examined the coverage of dimensions relating to the person, activity, participation and environmental factors, together with the operationalisation of interactions among components, AI-specific properties and everyday-life outcomes over time. The person-related dimension included both body functions and structures, when directly assessed, and personal characteristics and factors, such as goals, preferences, expectations, perceptions, motivation, choice and control. Although personal factors are not classified within the ICF through specific categories, they are recognised as relevant to the interaction between health conditions, functioning and context. The results concerning the coverage of these dimensions across the included evaluation approaches are presented in Table 2. Independent E/P/A coding across the seven comparative dimensions showed substantial agreement between the two researchers (Cohen’s κ = 0.81).

The matrix shows a markedly uneven distribution of the dimensions analysed across the included evaluation approaches. The person-related dimension was explicitly operationalised in 15 approaches and partially operationalised in one. The information collected concerned primarily personal factors, whereas body functions and structures were less frequently treated as distinct objects of evaluation. The most represented indicators included satisfaction, acceptance, trust, experience of interaction, expectations, perceived usefulness and behavioural responses. In *ATD PA* and *IPPA*, personal goals, preferences and individually prioritised problems formed explicit components of the evaluation. The *AI-enabled AT Framework* included choice and control, while *Value Preference Profiles* and *Ethical Compliance Quantification* used value preferences to examine automated actions. The person-related dimension was classified as partial only in *SHARA-WoZ* because, in the initial study, the perspective of older adults was represented through simulation and expert evaluation rather than through their direct involvement.

Activity was explicitly operationalised in nine approaches, partially represented in six and absent in one. It was assessed directly when an approach included tasks, scenarios, action analysis or the individualised identification of problems in everyday activities. In IPPA, problematic activities constituted the primary object of evaluation. In the *AI TEVV for Accessibility Framework*, *X-HAZOP*, *SHARA-WoZ*, *QUEAD* and the *Acceptance Test for Assistive Robots*, activities were represented through formally specified tasks or scenarios. In instruments centred on satisfaction, acceptance or psychosocial impact, activity was represented indirectly through indicators such as usefulness, effectiveness, performance or perceived benefit. *QUEST 2.0* did not collect distinct information on the activities performed.

Participation showed the most limited coverage. It was explicitly operationalised only in *ATD PA*, partially represented in eight approaches and absent in seven. In *IPPA*, participation could be included when the problems identified concerned involvement in life situations, whereas *PIADS* represented it indirectly through competence, adaptability, independence and willingness to engage in new situations. In frameworks addressing social, ethical or rights-related dimensions, participation was reflected through concepts such as inclusion, equity, autonomy, societal impact or quality of life, but was not assessed as a distinct domain.

Environmental factors were explicitly operationalised in six approaches, partially represented in nine and absent in one. Explicit coverage included support and milieu in *ATD PA*, accessibility and societal impact in the *AUSUS Evaluation Framework*, field-testing conditions in the *AI TEVV for Accessibility Framework*, *scenarios and stakeholders* in *X-HAZOP*, the organisation of care and the home environment in *UNRAQ*, and everyday contexts and care relationships in *Value Preference Profiles* and *Ethical Compliance Quantification*. In the other approaches, the environment was represented through more restricted elements, such as service provision, the experimental setting, the presence of professionals or interface characteristics. *PIADS* did not include distinct indicators of environmental factors.

Interaction between the components was explicitly operationalised in 13 approaches and partially operationalised in three. In approaches classified as explicit, characteristics of the person, technology, task or context were connected through formally specified mechanisms. In *ATD PA*, this relationship took the form of a matching process connecting personal characteristics, goals, activities, technology and environment. In *IPPA*, it connected the person’s priority problems with the intervention and the change subsequently assessed. In more recent frameworks and structured procedures, interaction was represented through testing sequences, use scenarios, analyses of deviations and risks, simulations, or comparisons between different levels of autonomy and system behaviour. *QUEAD*, the *Almere Model and the Acceptance Test for Assistive Robots* linked the person’s experience or reactions to specific tasks, control modes or robot behaviours.

Interaction was only partially operationalised in *QUEST 2.0*, *PIADS* and *PYTHEIA*. These instruments primarily collected overall judgements about the product, its functions, or the effects attributed to its use, without formally reconstructing how the person, activity, technology, and environment jointly contributed to the observed outcome.

An explicit relationship between components, however, did not imply complete coverage of all the dimensions analysed. Several approaches connected the person and technology, or the technology user, task and system behaviour, without including participation or environmental factors as distinct domains. Among the approaches included in this review, *ATD PA* was the only one to explicitly cover the person, activity, participation, and environment and to integrate them within a matching process. Even in this case, however, the approach did not provide an independent longitudinal measure of change across all four dimensions. Explicit operationalisation of interaction, therefore, indicated the presence of a formalised connection between components, rather than the completeness, quality or longitudinal capability of the evaluation.

The specific properties of artificial intelligence constituted an explicit and distinct object of evaluation in three approaches: the *AI-enabled AT Framework*, the *AI TEVV for Accessibility Framework and Value Preference Profiles* and *Ethical Compliance Quantification*. They were partially considered in *X-HAZOP* and *SHARA-WoZ*, respectively, through the risks associated with autonomy and comparisons between different modes of operation. In the remaining 11 approaches, the algorithmic component was not evaluated separately from the technology’s overall characteristics. This distribution shows that the formal representation of person–technology interaction does not necessarily entail examining the specific contribution of AI.

A subgroup comparison restricted to the five approaches developed specifically around AI-enabled or automated assistive systems yielded the same overall pattern. None explicitly operationalised participation, and none combined explicit coverage of person, activity, participation and environment with explicit evaluation of AI-specific properties and structured longitudinal assessment of everyday-life outcomes. The central finding of fragmentation across these dimensions therefore did not depend on the inclusion of established technology-agnostic assistive-technology instruments.

The outcomes most frequently assessed concerned satisfaction, acceptance, usability, perceived usefulness, experience of interaction, trust and willingness to use the technology. *IPPA* assessed changes in the difficulty of problems prioritised by the person, while *PIADS* addressed psychosocial impact through competence, adaptability and self-esteem. *ATD PA* evaluated the quality of the match and the conditions associated with technology use or abandonment. More recently developed frameworks and procedures also examined accessibility, technical quality, safety, privacy, equity, rights, ethical risks and the alignment between automated actions and the person’s preferences.

Everyday-life outcomes over time were explicitly operationalised only in *IPPA* and PIADS, were partially represented in 11 approaches, and were absent in three. In the approaches classified as partial, everyday life was addressed through scenarios, general judgements, expectations, periods of use or the possibility of field testing, without a structured assessment of changes in activity and participation. *SHARA-WoZ*, *QUEAD* and the *Acceptance Test for Assistive Robots* primarily assessed experience during or immediately after a controlled trial. Continuity of use, transfer of effects across environments and maintenance of outcomes over time were not systematically operationalised. Among the approaches included in this review, none combined explicit coverage of the person, activity, participation and environment with an evaluation of AI-specific properties and longitudinal monitoring of outcomes in everyday life situations.

## 4. Discussion

The 16 evaluation approaches identified in this review reveal a field characterised less by a shortage of evaluative resources than by fragmentation across disciplinary traditions [21,22]. The analysis was conducted at the level of evaluation approaches, drawing on the primary reference for each approach and, where available, on relevant validation or application publications. The contribution of this discussion is therefore to interpret the conceptual and methodological fragmentation observed across the included approaches as a defined theoretical problem rather than as a quantitative shortage of measures. Evaluation approaches developed within established assistive-technology assessment and outcome-evaluation traditions generally emerged from a paradigm that treats assistive technology as a stable and bounded object whose adoption, satisfaction and impact can be assessed after its introduction. *AI-enabled assistive technologies*, in contrast, place an adaptive, probabilistic and changing mediator at the centre of the relationship, whose behaviour depends on data, learning and conditions of use. The person-centred tradition [3,4] remains the appropriate conceptual basis for examining these technologies, but it requires an extension capable of accounting for an object that changes while it is being used.

This extension concerns matching models in the first instance. The *Matching Person and Technology model* [19], the *Human Activity Assistive Technology model* [20] and the outcome model developed by Fuhrer [18] interpret the success of an intervention as a fit between the characteristics of the person, technology, activity and environment, and connect this fit with the risk of device abandonment [59]. Some established instruments allow matching or outcomes to be reassessed after technology provision, but they generally treat the technology as a unitary product whose relevant characteristics can be evaluated through repeated overall judgements. *AI-enabled systems* complicate this assumption because their outputs and performance may change following software updates, personalisation, variations in input data or changes in environmental conditions. An initially adequate match may therefore deteriorate even when the device remains available and operational. The theoretical challenge is not simply to repeat the same assessment over time, but to reconceptualise matching as a dynamic relationship and to distinguish changes in the person, activity, or environment from those attributable to algorithmic behaviour, data drift, and variations in performance across groups and contexts [12,13].

A second issue concerns how a person with a disability is represented within an evaluation. The person-related dimension is almost always present and focuses primarily on satisfaction, acceptance, trust and perceived usefulness, while individual goals, the negotiation of delegated functions and the ability to correct or interrupt automation are represented less frequently. This configuration reflects the legacy of the instruments employed. The technology-acceptance tradition, exemplified by the *Almere Model*, measures intention to use and willingness to adopt a system—constructs designed to predict adoption rather than to describe the control exercised during use. Interpreting acceptance and trust as indicators of control over automation produces a conceptual shift that the literature on agency cautions against. Bandura [60] defines agency as the capacity to intentionally influence the course of events, while Limerick and colleagues [61] show that the sense of agency remains distinct from a favourable judgement of a system. Research on trust calibration in human–AI interaction supports this distinction by linking appropriate reliance to system comprehensibility and to the ability to verify and correct its operation [62]. Because automation intervenes in activities connected to identity and self-determination [14,15], agency should be treated as a distinct evaluative dimension, operationalised through the conditions required to understand, delegate, verify, correct, reject and interrupt automation, rather than inferred from an overall judgement of acceptance.

Participation and the environment are the two areas where the gap between the field’s theoretical foundations and available instruments is widest. The ICF interprets functioning as the outcome of the interaction between activity, participation, and contextual factors [17], whereas the review found that participation was operationalised as an independent domain in very few approaches and that the environment was inconsistently represented, often reduced to the testing setting. For *AI-enabled technologies*, this reduction has specific theoretical implications: lighting, noise, language, connectivity, data quality and configuration contribute to determining system performance itself, rather than merely providing the setting in which performance occurs [12,13,26]. The environment, therefore, functions as an active component of system functioning, consistently with the person–environment–tool model [63]. Incorporating it within an overall judgement of the device obscures the interaction between the person’s characteristics, contextual conditions and algorithmic behaviour on which equity and safety depend [13]. The rights-based perspective developed in consumer-led approaches—which link outcomes to the ICF domains of activity and participation and advocate measuring what matters to persons with disabilities [64]—offers a means of restoring participation as a primary outcome rather than deriving it from indicators of usefulness or competence.

The transition from the experience of interaction to an assistive outcome lies at the centre of the evaluation problem. The review found broad coverage of proximal and subjective outcomes—usability, acceptance and satisfaction—but more limited coverage of distal and ecological outcomes, namely sustained changes in activity and participation. This asymmetry reproduces the established distinction in assistive device outcome research between proximal and distal effects [18], as well as the taxonomy that distinguishes effectiveness, social significance, and subjective well-being [65]. AI adds an attribution problem to this distinction: an observed improvement may arise from algorithmic behaviour, the interface, the training provided, professional support, or the reorganisation of the context. Most instruments treat the technology as an undifferentiated whole, preventing the specific contribution of the algorithmic component from being isolated.

Across the approaches included in this review, two complementary evaluative traditions can be identified, although their integration remains partial. Frameworks for evaluating and governing intelligent systems—from the *NIST AI Risk Management Framework* [66] to *DECIDE-AI* [67] and *FUTURE-AI* [68], together with testing and risk-analysis approaches [26,29] addressing accessibility and assistive robotics—introduce system behaviour, life-cycle monitoring and failure modes into evaluation. Instruments developed within assistive-technology assessment instead provide stronger coverage of individual goals, matching, experience of use and outcomes in everyday life, but generally do not distinguish the algorithmic component from the technology as a whole. Some recent frameworks begin to connect these perspectives. However, among the approaches included in this review, none combined comprehensive coverage of the person, activity, participation and environment with AI-specific evaluation and structured longitudinal monitoring. The available approaches, therefore, indicate the need for a more systematic connection between technical performance and the consequences produced in meaningful activities and participation.

Based on the conceptual and methodological gaps identified across the included approaches, this review proposes assistive validity as a higher-order evaluative criterion rather than as a psychometric property or an already validated measurement construct.

It refers to the extent to which the performance of an intelligent system translates, for a particular person with a disability and under specific environmental conditions, into support that is accessible, controllable, safe and meaningful for activity and participation. This working definition is theoretically informed by Wolf’s [69] concept of social validity, centred on the significance of goals, procedures and effects for those directly concerned, and by Messick’s [70] consequential account of validity, which draws attention to the implications associated with the interpretation and use of assessment results. These perspectives converge with the outcome model developed by Fuhrer [18] and the taxonomy proposed by Jutai [65], while the operational content and empirical properties of assistive validity remain to be established.

Assistive validity is not intended as a substitute for person–technology fit, social validity, ecological validity, usability, accessibility or AI trustworthiness. Rather, it addresses a distinct evaluative question: whether the technical and algorithmic properties of an AI-enabled assistive technology translate, for a particular person and in a particular context, into meaningful support for activity and participation while preserving the person’s agency. Person–technology fit concerns the correspondence between the person, the technology and the conditions of use; social validity concerns the significance and acceptability of goals, procedures and outcomes for those directly concerned; and ecological validity concerns the extent to which an evaluation reflects functioning under real-world conditions. Usability and accessibility address whether the system can be used effectively and accessed by the intended user, whereas AI trustworthiness concerns properties such as robustness, safety, transparency and fairness. Assistive validity does not replace any of these dimensions. Its specific function is to examine whether they converge in the production of an assistive outcome that is meaningful for the person’s goals, activities and participation.

The primary evaluative referent of assistive validity is the person with a disability in relation to the goals, activities and participation that the technology is intended to support. Other actors—including clinicians, caregivers, service providers, designers, engineers and accessibility specialists—may contribute evidence at different stages, but their assessments do not replace the person’s perspective on the assistive consequences of technology use. Assistive validity is therefore conceived as a cross-stage criterion rather than as an assessment confined to a single point in development. Relevant evidence may be generated during design, selection, technical testing and early deployment, whereas its strongest demonstration requires evaluation in real-world contexts and, where the assistive function warrants it, follow-up over time to establish whether technically adequate performance is translated into sustained support for activity, participation and agency.

From this perspective, five propositions delimit the proposed construct. First, technical or algorithmic performance may be necessary for assistive benefit, but it is not sufficient to establish assistive validity. Second, assistive validity is relational and context-dependent rather than an intrinsic property of a technology. Third, it depends on the extent to which system performance supports goals, activities and participation that are meaningful for the person. Fourth, agency—including the possibility of understanding, influencing, correcting, rejecting or interrupting automated actions—is an integral component of assistive validity rather than an ancillary outcome. Fifth, the strongest evidence of assistive validity requires demonstration that these relationships are maintained in real-world use and, where relevant, over time.

Within this framework, accuracy, robustness, usability and acceptance remain relevant but individually insufficient evaluative dimensions, whose assistive significance depends on the function performed, the context of use and the consequences of error. An inaccurate classification carries different implications when a system recommends recreational content, generates a message, describes an obstacle or supports mobility. Assistive validity therefore requires these properties to converge in a meaningful and sustainable change in the possibilities for action available to a person with a disability. It points to a modular, longitudinal evaluation architecture, applicable from selection through follow-up, rather than a single comprehensive scale. The calibration of standardised tests, ecological trials and participatory procedures according to the assistive function and level of risk applies the risk-based logic of AI risk management frameworks to the assistive technology field [66,68].

Established instruments can also form part of this architecture, provided that their properties are critically reassessed. The *Rasch analysis of QUEST 2.0* conducted by Caronni [56] identified a marked ceiling effect, low reliability and a lack of measurement invariance across device types, making the total score problematic for comparisons between different technologies. An established procedural basis may therefore coexist with measurement limitations that remain implicit when evaluation treats the device as an undifferentiated whole. This example also illustrates why evidence derived from subsequent validation or re-evaluation studies must be considered separately from the original development publication when characterising an approach. A similar issue arises in human–robot interaction, where the instability and limited comparability of the metrics employed constrain synthesis across studies [71].

From this perspective, ethical dimensions and rights constitute internal components of assistive validity rather than an additional layer placed alongside technical evaluation. The automation of functions related to care, communication, and mobility affects self-determination, privacy, and responsibility [15,72,73], while alignment between automated actions and a person with a disability’s value preferences becomes a substantive evaluation criterion [28]. Relating these aspects to the significance of effects for the person, according to the logic of social validity, keeps equity, safety and control within everyday life rather than confining them to a separate technical assessment.

Translating assistive validity into an empirically testable evaluation architecture will require staged development rather than the construction of a single additional scale. An initial phase should establish content relevance through co-design with persons with disabilities and the other actors involved in assistive-technology design, provision and evaluation, followed by specification of the domains, indicators and relationships that operationalise the proposed criterion. Subsequent studies should examine the feasibility and usability of the resulting evaluation architecture in different assistive contexts and, where quantitative measures are incorporated, assess their reliability and measurement properties. Convergent and discriminant evaluation should then determine whether assistive validity provides information that is not already captured by technical performance, usability, accessibility, acceptance or related constructs. Finally, longitudinal ecological studies should examine whether the proposed architecture is able to account for sustained changes in meaningful activity, participation and agency, as well as continued technology use or abandonment, under real-world conditions. This staged programme would provide the empirical basis needed to determine whether assistive validity can progress from an interpretive criterion to an operationally useful evaluation framework.

The proposed interpretation has several limitations. Despite the structured search and source-selection process, the set of approaches identified may have been influenced by the databases searched, the restriction to English-language publications and the requirement that approaches be sufficiently formalised for comparative analysis. In particular, CINAHL and the ACM Digital Library were not searched directly. Their omission may have resulted in the underrepresentation of evaluation approaches reported primarily within allied-health, rehabilitation, human–computer interaction or accessibility venues that are less consistently indexed in the databases included in this review. Accordingly, the review should be interpreted as a critical synthesis of the approaches identified through the adopted search architecture rather than as an exhaustive mapping of all evaluation approaches available across these fields.

The review was not prospectively registered. Although the research questions, eligibility criteria and coding rules are reported transparently, the absence of preregistration limits the possibility of distinguishing completely between decisions specified before screening and refinements developed iteratively during the interpretive synthesis, and therefore leaves some potential for post hoc analytical decisions. The analysis was conducted at the level of evaluation approaches rather than at the level of individual studies. For each approach, the primary reference and, where available, relevant validation or application publications were used to characterise its methodological properties, purposes, populations, technologies and evaluated dimensions. The number, type and maturity of the supporting publications therefore varied across approaches. Consequently, classification of a property as not documented indicates that relevant evidence was not identified in the sources examined; it does not demonstrate that the property is intrinsically absent from the approach. Grouping development, validation and application publications under a single evaluation approach may also obscure differences across specific versions, populations, technologies or contexts of use. The rapid evolution of the field makes the selection necessarily provisional. The temporal distribution reported in the review reflects when the approaches were introduced and captured using the adopted criteria, rather than reconstructing the field’s overall historical development. Classifying dimensions as explicit, partial or absent describes their degree of operationalisation but does not establish the methodological quality of every study or application. Several recent frameworks are also supported only by preliminary evidence, and assistive validity requires empirical investigation to determine whether it can be translated into procedures applicable in service-delivery and everyday-life contexts.

## 5. Conclusions

Across the 16 evaluation approaches identified in this review, no single instrument, framework or structured procedure integrates the evaluation of system behaviour with the goals and experiences of persons with disabilities, activities, participation, environmental conditions and longitudinal everyday-life outcomes. The included approaches are nevertheless potentially complementary and, taken together, indicate the need for a modular and longitudinal evaluation architecture spanning needs assessment, technical verification, use and follow-up.

On the basis of the conceptual and methodological gaps identified, this review proposes assistive validity as a higher-order evaluative criterion linking technical performance, agency and meaningful outcomes in everyday life, rather than as an already validated measurement construct. Future research should operationalise and empirically examine this criterion by combining measures of individual goals, person–technology matching, and experience of use with procedures that address algorithmic quality, accessibility, safety, equity, and control. Longitudinal studies in real-world contexts are also required to establish whether, for whom and under which environmental and organisational conditions *AI-enabled assistive technologies* sustainably expand opportunities for action, choice and participation.

## Figures and Tables

**Table 1:** Identifying characteristics, reference publications, selected validation and/or application studies, purposes, application fields and summary evidence profiles of the included evaluation approaches ^1^.

Year	Approach	Reference Paper	Validation and/or Application Papers	Type	Primary Purpose and Stage	Target Technology and Application Population or Field	Summary Evidence Profile
2026	*AI TEVV for Accessibility Framework*	Waters, G. (2026), *AI testing, evaluation, verification and validation for accessibility: A comprehensive framework* [26]	Illustrative case studies are presented within Waters (2026) [26]; no independent validation or application study of the complete framework was identified.	Procedural framework	Testing and monitoring throughout the system life cycle	AI systems and AI-enabled technologies evaluated for accessibility, including assistive applications	Strong conceptual and methodological specification; integrated empirical application, reproducibility and feasibility of the complete framework remain undocumented
2026	*SHARA-WoZ*	Cubero, G.; Villa, L.; Mondéjar, T.; Hervás, R. (2026), *SHARA-WoZ: A multistakeholder framework to evaluate socially assistive robots through Wizard-of-Oz methods* [27]	The initial expert-based laboratory application is reported within Cubero et al. (2026) [27]; no independent validation or real-world application paper was identified.	Multistakeholder procedural framework	Prototyping and comparison of manual, semi-autonomous and autonomous modes	Socially assistive robots intended for older adults	Initial laboratory application documented; direct involvement of the intended older users, real-world feasibility and procedural reproducibility remain undocumented
2025	*AI-enabled AT Framework*	Silvera-Tawil, D.; Higgins, L.; Packer, K.; et al. (2025), *AI-enabled AT Framework: A principles-based approach to emerging assistive technology* [8]	No independent empirical validation or application of the complete framework was identified.	Principle-based framework	Design, selection, implementation and review	*AI-enabled assistive technologies* across application domains and disability groups	Content relevance is supported by participatory development; empirical application, procedural reproducibility and feasibility of the complete framework remain undocumented
2025	*Value Preference Profiles and Ethical Compliance Quantification*	Buhr, E.; Welsch, J.; Shaukat, M.S. (2025), *Value preference profiles and ethical compliance quantification: A new approach for ethics by design in technology-assisted dementia care* [28]	Empirical preference-profile development and simulation-based application are reported in the reference paper. No independent external validation was identified.	Ethics-by-design and simulation procedure	Development of value preference profiles and prospective evaluation of automated actions	Automated assistive systems intended for persons with dementia	Empirical preference profiles and simulation-based application documented; external validation, implementation in routine practice and real-world feasibility remain limited
2025	*X-HAZOP*	Menon, C.; Rainer, A.; Holthaus, P.; Moros Español, S.; Lakatos, G. (2025), *X-HAZOP: A family of techniques for ethical hazard analysis of assistive robots* [29]	Menon, C.; Rainer, A.; Holthaus, P.; Lakatos, G.; Carta, S. (2024), *EHAZOP: A proof-of-concept ethical hazard analysis of an assistive robot* [30] The 2025 paper also reports workshop-based applications.	Participatory structured procedure	Prospective analysis of ethical and social risks	Assistive robots used in domestic, healthcare and care contexts	Proof-of-concept and workshop-based applications documented; independent reproducibility and performance in real-world use remain unestablished
2022	*Robot Era Inventory*	Bevilacqua, R.; Di Rosa, M.; Riccardi, G.R.; et al. (2022), *Design and development of a scale for evaluating the acceptance of social robotics for older people: The Robot Era Inventory* [31]	Preliminary psychometric testing is included in Bevilacqua et al. (2022) [31]. No independent validation or application study using the completed inventory was identified.	Standardised self-report scale	Evaluation following presentation of or interaction with the system	Socially assistive robots intended for older adults	Preliminary validity and reliability evidence from a small sample; independent validation, cross-cultural adaptation, responsiveness and longitudinal evidence remain undocumented
2021	*AUSUS Evaluation Framework*	Iglesias, A.; García, J.; García-Olaya, A.; et al. (2021), *Extending the evaluation of social assistive robots with accessibility indicators: The AUSUS Evaluation Framework* [32]	The reference paper includes application to the CLARC robot. Jerez, A.; Iglesias, A.; Pérez-Lorenzo, J.M.; et al. (2024), *A user-centered evaluation of two socially assistive robots integrated in a retirement home* [33], provide a subsequent real-world application.	Multidimensional framework	Design, experimentation and field evaluation	Socially assistive robots used in healthcare and care settings	Initial single-system application and a subsequent retirement-home application documented; procedural reproducibility, use across broader populations and longitudinal outcome capability remain insufficiently established
2021	*UNRAQ*	Tobis, S.; Neumann-Podczaska, A.; Kropińska, S.; Suwalska, A. (2021), *UNRAQ—A questionnaire for the use of a social robot in care for older persons: A multi-stakeholder study and psychometric properties* [34]	Tobis, S.; Piasek-Skupna, J.; Neumann-Podczaska, A. et al. (2023), *The effects of stakeholder perceptions on the use of humanoid robots in care for older adults: Postinteraction cross-sectional study* [35]. Trainum et al. (2026) *report psychometric testing of N-UNRAQ, a modified nursing version* [36],	Standardised multistakeholder questionnaire	Preliminary assessment of needs, requirements and expectations	Social and assistive robots intended for older adults	Reliability, feasibility and postinteraction application are documented; validity in relation to subsequent routine use and predictive capacity for longer-term experience remain only partially supported
2020	*Acceptance Test for Assistive Robots*	Martín Rico, F.; Rodríguez-Lera, F.J.; Matellán Olivera, V. (2020), *An Acceptance Test for Assistive Robots* [37]	The controlled feasibility trial involving persons with dementia is reported in the reference paper; no independent replication or external application study was identified.	Structured observational procedure	Evaluation during and immediately after interaction	Socially assistive robots intended for persons with dementia	Feasibility demonstrated in a brief controlled trial; inter-observer reproducibility, independent replication and broader empirical application remain undocumented
2017	*QUEAD*	Schmidtler, J.; Bengler, K.; Dimeas, F.; Campeau-Lecours, A. (2017), *A Questionnaire for the Evaluation of Physical Assistive Devices (QUEAD): Testing usability and acceptance in physical human–robot interaction* [38]	Two preliminary device studies supporting validity and reliability are reported in Schmidtler et al. (2017) [38]. No independent validation involving persons with disabilities and an explicitly AI-enabled assistive system was identified.	Structured questionnaire	Evaluation during or after tasks and across different configurations	Physical assistive devices and robotic manipulators	Preliminary validity and reliability evidence from restricted samples; independent validation, responsiveness, cross-cultural adaptation and everyday life use remain undocumented
2016	*PYTHEIA*	Koumpouros, Y.; Papageorgiou, E.; Karavasili, A.; Koureta, F. (2016), *PYTHEIA: A scale for assessing rehabilitation and assistive robotics* [39]	Koumpouros et al. (2017), *User evaluation of the MOBOT rollator type robotic mobility assistive device* [40]; Koumpouros et al. (2020), *Assessment of an intelligent robotic rollator implementing navigation assistance in frail seniors* [41]; Moustris et al. (2021), *The i-Walk lightweight assistive rollator: First evaluation study* [42]	Structured self-report scale	Evaluation immediately after testing	Rehabilitation and assistive robots and devices	Preliminary validity and reliability evidence and several robotic applications are documented; responsiveness, cross-cultural adaptation and routine longitudinal use remain undocumented
2010	*Almere Model*	Heerink, M.; Kröse, B.; Evers, V.; Wielinga, B.J. (2010), *Assessing acceptance of assistive social agent technology by older adults: The Almere Model* [43]	Heerink et al. (2009), Influence of social presence on acceptance of an assistive social robot and screen agent by elderly users [44]; Fracasso et al. (2022), cross-national application concerning older persons’ acceptance of social robots in assisted-living contexts [45].	Acceptance model and questionnaire	Evaluation before, after or during periods of use	Socially assistive robots and intelligent agents intended primarily for older adults	Validity, reliability, adaptation, feasibility and application across multiple studies are documented; formal responsiveness and capacity to measure longer-term change remain unestablished
2002	*IPPA*	Wessels, R.; Persson, J.; Lorentsen, Ø.; et al. (2002), *IPPA: Individually Prioritised Problem Assessment* [46]	Bemelmans et al. (2015), application to the PARO robot in psychogeriatric care [47]; van den Heuvel, R.J.F.; Lexis, M.A.S.; de Witte, L.P. (2020), *ZORA robot-based interventions to achieve therapeutic and educational goals in children with severe physical disabilities* [48].	Structured individualised interview	Initial identification and reassessment of priority problems	General assistive technologies, including documented applications to robotic systems	Validity and responsiveness, together with applications to robotic systems, are documented; reliability and formal cross-cultural adaptation remain less extensively supported
2002	*PIADS*	Jutai, J.; Day, H. (2002), *Psychosocial Impact of Assistive Devices Scale (PIADS)* [49]	Demers et al. (2002), Canadian-French translation and preliminary psychometric evaluation [50]; Oldford et al. (2022), application to robotic assistive technology for adolescents with neuromuscular disease [51]; Pousada García et al. (2022), *MATCH protocol involving AI-enabled robotic assistive technology* [52]	Standardised self-report scale	Evaluation following introduction and at follow-up	General assistive technologies, including robotic applications and protocolled use with AI-enabled robotic systems	Validity, reliability and cross-cultural adaptation are documented; responsiveness evidence remains partial and inconsistent, while completed empirical applications to explicitly *AI-enabled systems* remain limited
2002	*QUEST 2.0*	Demers, L.; Weiss-Lambrou, R.; Ska, B. (2002), *The Quebec User Evaluation of Satisfaction with Assistive Technology (QUEST 2.0): An outcome measure for assistive technology devices* [53]	Demers et al. (2002), reliability, validity and applicability in adults with multiple sclerosis [54]; Wessels and de Witte (2003), Dutch validation [55]; Koumpouros et al. (2017), application to the MOBOT robotic rollator [40]; Caronni et al. (2023), Rasch analysis [56].	Standardised questionnaire	Evaluation following provision and at follow-up	General assistive technologies, including documented applications to intelligent and robotic systems	Validity, reliability, cross-cultural adaptation and feasibility are documented; responsiveness remains insufficiently established, and Rasch evidence raises concerns about total-score reliability and comparability across device types
2001	*ATD PA*	Scherer, M.J.; Cushman, L.A. (2001), *Measuring subjective quality of life following spinal cord injury: A validation study of the Assistive Technology Device Predisposition Assessment* [57]	Scherer and Craddock (2002), description of the MPT assessment process [19]; Koumpouros et al. (2017), Greek translation and validation [58]; Koumpouros et al. (2017), application to the MOBOT robotic rollator [40]; Pousada García et al. (2022), *MATCH protocol involving AI-enabled robotic assistive technology* [52]	Standardised assessment instrument	Needs assessment, matching and reassessment	General assistive technologies, including documented applications to robotic systems	Validity, reliability, cross-cultural adaptation, feasibility and robotic application are documented; responsiveness has not been specifically examined, and evidence from explicitly AI-enabled applications remains limited and partly protocol-based

^1^ The reference paper is the primary publication used to identify and characterise the evaluation approach. Validation and/or application papers comprise selected sources used to support the evidence profile and inclusion of the approach in the review and are not intended as an exhaustive bibliography. “Not identified” indicates that relevant evidence was not identified in the sources examined for this review and does not demonstrate that such evidence does not exist.

**Table 2:** Coverage and operationalisation of the dimensions analysed across the included evaluation approaches ^2^.

Year	Approach	Person	Activity	Participation	Environment	Interaction Among Components	AI-Specific Properties	Everyday-Life Outcomes Over Time
2026	*AI TEVV for Accessibility Framework*	E	E	P	E	E	E	P
2026	*SHARA-WoZ*	P	E	A	P	E	P	A
2025	*AI-enabled AT Framework*	E	P	P	P	E	E	P
2025	*Value Preference Profiles and Ethical Compliance Quantification*	E	E	P	E	E	E	P
2025	*X-HAZOP*	E	E	P	E	E	P	P
2022	*Robot Era Inventory*	E	P	A	P	E	A	P
2021	*AUSUS Evaluation Framework*	E	P	P	E	E	A	P
2021	*UNRAQ*	E	E	P	E	E	A	P
2020	*Acceptance Test for Assistive Robots*	E	E	A	P	E	A	A
2017	*QUEAD*	E	E	A	P	E	A	A
2016	*PYTHEIA*	E	P	A	P	P	A	P
2010	*Almere Model*	E	P	A	P	E	A	P
2002	*IPPA*	E	E	P	P	E	A	E
2002	*PIADS*	E	P	P	A	P	A	E
2002	*QUEST 2.0*	E	A	A	P	P	A	P
2001	*ATD PA*	E	E	E	E	E	A	P
	**E/P/A distribution**	**15/1/0**	**9/6/1**	**1/8/7**	**6/9/1**	**13/3/0**	**3/2/11**	**2/11/3**

^2^ Notes: E = explicit; P = partial; A = absent. For the dimensions relating to the person, activity, participation and environment, coverage was classified as explicit when the dimension constituted an independent domain, item set or procedural stage; partial when it was addressed through individual items, indirect indicators or proxies; and absent when it was not operationalised. For the person-related dimension, explicit coverage required the intended users’ characteristics, goals, experiences or responses to be directly elicited or independently operationalised; coverage was classified as partial when the person’s perspective was represented mainly through proxy respondents, simulation, expert judgement or inferred characteristics. For interaction among components, E indicates that a formalised mechanism connects the technology with one or more other components—person, activity or task, participation, or environment—through matching, structured scenarios, task-based trials, testing sequences or relational models; P indicates that the relationship can be inferred only from overall judgements concerning the product, its functions or its effects; A indicates that the components are assessed separately or that no relationship among them is described. For AI-specific properties, E indicates that properties such as output quality, accuracy, robustness, bias, autonomy or alignment of automated actions are evaluated as distinct objects; P indicates that only selected manifestations of automation, modes of operation or associated risks are considered; A indicates that the algorithmic component is not distinguished from the overall characteristics of the technology. For everyday-life outcomes over time, E indicates that outcomes are assessed after a period of use in everyday-life contexts through a structure designed to capture change or follow-up; P indicates that everyday life or temporality is addressed through scenarios, general judgements, periods of use or the possibility of field testing, without a structured assessment of changes in activity and participation; A indicates that evaluation is limited to a controlled or immediate trial.

## Data Availability

No new data were created or analysed in this study. Data sharing is not applicable to this article.

## Supplementary Materials

The following supporting information can be downloaded at https://www.mdpi.com/article/10.3390/healthcare14182900/s1. Table S1: Database-specific search strategies and records retrieved; Table S2: Methodological characteristics and evidence profiles of the included evaluation approaches; Table S3: Target populations, technologies, AI components and AI-specific properties evaluated across the included approaches.

## Abbreviations

The following abbreviations are used in this manuscript:

AI	Artificial Intelligence
AT	Assistive Technology
ATD PA	Assistive Technology Device Predisposition Assessment
AUSUS	Accessibility, Usability, Social acceptance, User experience and Societal impact
DECIDE-AI	Developmental and Exploratory Clinical Investigations of DEcision support systems driven by Artificial Intelligence
FUTURE-AI	Fairness, Universality, Traceability, Usability, Robustness and Explainability in Artificial Intelligence
ICF	International Classification of Functioning, Disability and Health
IEEE	Institute of Electrical and Electronics Engineers
IPPA	Individually Prioritised Problem Assessment
MEDLINE	Medical Literature Analysis and Retrieval System Online
NIST	National Institute of Standards and Technology
PIADS	Psychosocial Impact of Assistive Devices Scale
PYTHEIA	Psychometric Scale to Assess the Satisfaction of Users with Assistive Technology
QUEAD	Questionnaire for the Evaluation of Physical Assistive Devices
QUEST 2.0	Quebec User Evaluation of Satisfaction with Assistive Technology, version 2.0
TEVV	Testing, Evaluation, Verification and Validation
UNICEF	United Nations Children’s Fund
UNRAQ	Users’ Needs, Requirements and Abilities Questionnaire
WHO	World Health Organization
WIPO	World Intellectual Property Organization
WoZ	Wizard of Oz
X-HAZOP	X-Hazard and Operability Analysis
