# Supplementary material for: From Technical Performance to Assistive Validity: A Critical Review of Evaluation Approaches for AI-Enabled Assistive Technology
Source: Healthcare (Basel). 2026 Sep 8;14(18):2900. doi: 10.3390/healthcare14182900 (PMC13606492; doi:10.3390/healthcare14182900)
Supplement: Supplementary file 1 [file healthcare-14-02900-s001.zip › healthcare-4505372-supplementary.pdf]

Supplementary Materials

Table S1: Database-specific search strategies and records retrieved; Table S2: Methodological characteristics and evidence profiles of the included evaluation approaches; Table S3: Target populations, technologies, AI components and AI-specific properties evaluated across the included approaches.

Table S1. Database-specific search strategies and records retrieved[1].

Database	Search period and final date	Fields searched	Limits and filters	Complete search strategy	Records retrieve d
IEEE Xplore	January-June 2026; final search conducted on 30 June 2026	All Metadata	No publication-date restrictions; all document types included	("All Metadata":"assistive technology" OR "assistive technologies" OR "assistive device" OR "assistive devices" OR "assistive robot" OR "assistive robots" OR "socially assistive robot" OR "socially assistive robots" OR "AI-enabled assistive technology" OR "accessibility technology" OR "accessibility technologies") AND ("artificial intelligence" OR "machine learning" OR "deep learning" OR "computer vision" OR "natural language processing" OR "large language model" OR "large language models" OR "generative AI" OR "intelligent system" OR "intelligent systems" OR "adaptive system" OR "adaptive systems") AND ("evaluation instrument" OR "measurement instrument" OR checklist OR framework OR protocol OR questionnaire OR scale OR usability OR acceptance OR trust OR explainability OR safety OR outcome OR assess OR evaluat) AND (disab OR impair OR rehabilitation OR "older adult" OR "older adults" OR dementia OR blind OR deaf OR aphasia OR "augmentative and alternative communication")	1,826
MEDLINE/PubM ed	January-June 2026; final search conducted on 30 June 2026	Title/Abstract [tiab]	No publication-date or document-type restrictions	((("assistive technology"[tiab] OR "assistive technologies"[tiab] OR "assistive device*"[tiab] OR "assistive system*"[tiab] OR "assistive robot*"[tiab] OR "socially assistive robot*"[tiab] OR "AI-enabled assistive technolog*"[tiab] OR "accessibility technolog*"[tiab]) AND ("artificial intelligence"[tiab] OR "machine learning"[tiab] OR "deep learning"[tiab] OR "computer vision"[tiab] OR "natural language processing"[tiab] OR "large language model*"[tiab] OR "generative AI"[tiab] OR "intelligent system*"[tiab] OR "adaptive system*"[tiab])) AND ("evaluation instrument*"[tiab] OR "measurement instrument*"[tiab] OR scale*[tiab] OR questionnaire*[tiab] OR framework*[tiab] OR protocol*[tiab] OR checklist*[tiab] OR evaluat*[tiab] OR assess*[tiab] OR outcome*[tiab] OR usabil*[tiab] OR	349

[1] The search strategies were developed and progressively refined between January and June 2026, and the final searches were completed on 30 June 2026. No publication-date restrictions were applied. The IEEE Xplore results comprised 1,534 conference papers, 260 journal articles, 18 magazine articles, 11 early-access articles and three book chapters. Backward and forward citation searching and manual reference-list inspection were also conducted, but the search report does not provide a separate record count for these supplementary procedures; the total of 2,433 refers to the records retrieved from IEEE Xplore, MEDLINE/PubMed and Scopus.

Database	Search period and final date	Fields searched	Limits and filters	Complete search strategy	Records retrieved
				accept*[tiab] OR trust[tiab] OR explainab*[tiab] OR safety[tiab])) AND (disab*[tiab] OR impair*[tiab] OR rehabilitation[tiab] OR "older adult*"[tiab] OR dementia[tiab] OR blind*[tiab] OR deaf*[tiab] OR aphasia[tiab] OR "augmentative and alternative communication"[tiab])	
Scopus	January-June 2026; final search conducted on 30 June 2026	Title, Abstract and Keywords (TITLE-ABS-KEY)	No publication-date or document-type restrictions	TITLE-ABS-KEY("assistive technology" OR "assistive technologies" OR "assistive device" OR "assistive robot" OR "socially assistive robot" OR "AI-enabled assistive technology" OR "accessibility technology") AND TITLE-ABS-KEY("artificial intelligence" OR "machine learning" OR "deep learning" OR "computer vision" OR "natural language processing" OR "large language model" OR "generative AI" OR "intelligent system" OR "adaptive system") AND TITLE-ABS-KEY(evaluat* OR assess* OR validation OR validity OR reliability OR psychometric* OR metric* OR measure* OR benchmark* OR questionnaire OR survey OR scale OR framework OR checklist OR protocol OR usability OR acceptability OR acceptance OR trust OR explainability OR transparency OR safety OR "human factors" OR "user experience" OR UX) AND TITLE-ABS-KEY(disab* OR impair* OR rehabilitation OR "older adult" OR dementia OR blind* OR deaf* OR aphasia OR "augmentative and alternative communication")	258
				Total records identified through database searching	2,433

Table S2[2]**.** Methodological characteristics and evidence profiles of the included evaluation approaches[3]**.**

---

[2] **Supplementary Note S1. Decision rules for evidence and dimensional coding**

The classifications used in the review were intended to describe the availability and degree of operationalisation of evidence rather than to rank the methodological quality of individual studies.

Evidence-profile coding (D/PD/ND/NA). A property was coded as documented (D) when at least one publication directly evaluated the property using a clearly described method and reported results relevant to that property. It was coded as partially documented (PD) when the available evidence was indirect, preliminary, restricted to a small or specific sample, limited to only part of the approach, derived mainly from expert or stakeholder judgement, or insufficient to establish the property across the complete approach. Not documented (ND) indicated that evidence directly addressing the property was not identified in the publications examined for the review. It did not imply that the property was intrinsically absent or that relevant evidence could not exist elsewhere. Not applicable (NA) was used when a property was not methodologically appropriate to the type or purpose of the evaluation approach.

For measurement instruments, validity, reliability, responsiveness, cross-cultural adaptation and feasibility were considered separately. Repeated administration for stability or use in a pre–post study was not treated as evidence of responsiveness unless the study explicitly examined the instrument's ability to detect change. International or cross-cultural use was not coded as cross-cultural adaptation unless a formal translation, adaptation or validation procedure was documented.

For frameworks and structured procedures, psychometric properties were not transferred from instruments incorporated within them. These approaches were instead classified according to development transparency and content relevance, empirical application of the complete approach, procedural reproducibility and feasibility.

Use of secondary validation and application publications. Evidence reported in validation or application studies was considered when it directly informed the property being classified. Such evidence was attributed to the evaluation approach only when the study examined the relevant instrument, framework or procedure in a form sufficiently consistent with the approach under review. Evidence concerning a modified derivative, an isolated component or an incorporated instrument was not automatically transferred to the complete approach.

Differences across publications. When publications relating to the same approach provided differing or incomplete evidence, the code represented the overall documentary profile rather than the most favourable individual finding. A D classification required direct and sufficiently interpretable evidence for the property; where evidence was restricted, inconsistent or only partially transferable across versions, populations or contexts, PD was assigned.

Dimensional coding (E/P/A). A dimension was coded as explicit (E) when it constituted an independent domain, item set, judgement criterion or procedural stage within the approach. Partial (P) was assigned when the dimension was represented through individual items, indirect indicators, proxies or inferred consequences without being independently operationalised. Absent (A) indicated that the dimension was not operationalised in the approach as examined.

For the person-related dimension, E required direct elicitation or independent operationalisation of the intended user's characteristics, goals, preferences, experiences or responses; proxy respondents, simulations, expert judgements or inferred characteristics were coded as P when they represented the person's perspective indirectly. For interaction among components, E required a formally described mechanism connecting the technology with the person, activity or task, participation or environment; P was used when the relationship could only be inferred from global judgements concerning the technology or its effects. For AI-specific properties, E required properties such as output quality, accuracy, robustness, bias, autonomy or alignment of automated actions to be evaluated as distinct objects; P indicated that only selected manifestations of automation, operating modes or associated risks were considered. For everyday-life outcomes over time, E required a structured assessment designed to capture change or follow-up after a period of use in everyday-life contexts; P indicated that everyday life or temporality was represented through scenarios, general judgements, periods of use or possible field testing without structured assessment of change in activity or participation.

Borderline classifications were discussed by the two researchers with reference to these decision rules and the publications supporting the evaluation approach. Final codes were assigned by consensus following independent coding.

[3] Evidence classification: D = documented; PD = partially documented; ND = not documented; NA = not applicable. Psychometric validity, reliability and responsiveness were appraised only for measurement instruments. Frameworks and structured procedures were appraised separately in relation to development transparency and content relevance, empirical application of the complete approach, procedural reproducibility and feasibility. Evidence relating to instruments incorporated within a framework or procedure was not transferred to the complete approach.

Panel A. Standardised and individualised measurement instruments.

Year	Approach	Persons involved and mode of administration	Stage or timing of administration	Validity	Reliability	Responsiveness	Cross-cultural adaptation	Feasibility
2022	Robot Era Inventory	Self-report instrument completed by older adults following presentation of or interaction with a socially assistive robot	Immediately following presentation or interaction	PD—preliminary evidence from a small sample	PD—preliminary evidence from a small sample	ND	ND	PD—initial administration documented, but routine use has not been established
2021	UNRAQ	Multistakeholder questionnaire involving groups concerned with the needs, requirements and expectations associated with social and assistive robots for older adults	Preliminary needs and requirements assessment, before implementation or extended use	PD—limited evidence in relation to subsequent experience with the robot	D	NA—not designed primarily to measure change over time	A modified nursing version of UNRAQ has undergone psychometric testing in a different national context. Because N-UNRAQ represents a modified derivative rather than a direct cross-cultural adaptation of the original questionnaire, this evidence was not coded as formal cross-cultural adaptation of UNRAQ	D
2017	QUEAD	Structured questionnaire completed by users during or following tasks performed with physical	During or immediately after task performance; may compare	PD—preliminary evidence from restricted samples	PD—preliminary evidence from	ND	ND	PD—documented mainly in controlled testing conditions

Year	Approach	Persons involved and mode of administration	Stage or timing of administration	Validity	Reliability	Responsiveness	Cross-cultural adaptation	Feasibility
		assistive devices or robotic arms	different system configurations		restricted samples			
2016	PYTHEIA	Structured self-report scale completed by users testing rehabilitation or assistive robots and devices	Immediately after interaction or system testing	PD—preliminary evidence	PD—preliminary evidence	ND	ND	PD—applications documented mainly in experimental settings
2010	Almere Model	Questionnaire administered to older adults before, during or after interaction with assistive social robots or intelligent agents	Before interaction, immediately after interaction or following a period of use	D—structural and predictive validity documented	D	PD—repeated administration documented, but formal responsiveness remains limited	D	D
2002	IPPA	Individualised interview in which assistive technology users identify and prioritise problems in activities considered important	Initial assessment followed by reassessment after provision or use of the technology	D	ND—consistent evidence was not identified	D	ND—international use is documented, but consistent formal adaptation procedures were not identified	D
2002	PIADS	Standardised self-report scale completed by assistive technology users	Following introduction of the technology and at follow-up	D	D	PD—change has been examined, but evidence is not consistent across applications	D	D
2002	QUEST 2.0	Standardised questionnaire completed by assistive technology users, addressing satisfaction with the device and service provision	Following provision and, where applicable, at follow-up	D	D	ND—formal evidence of responsiveness has not been consistently established	D	D
2001	ATD PA	Standardised assessment involving the person with a disability within needs	Before selection or provision and	D	D	ND—responsiveness has not been	D	D

Year	Approach	Persons involved and mode of administration	Stage or timing of administration	Validity	Reliability	Responsiveness	Cross-cultural adaptation	Feasibility
		assessment, technology matching and reassessment	during subsequent reassessment			specifically examined		

Panel B. Frameworks and structured procedures.

Year	Approach	Type	Persons involved and operational format	Primary stage or timing	Development transparency and content relevance	Empirical application of the complete approach	Procedural reproducibility	Feasibility
2026	AI TEVV for Accessibility Framework	Procedural framework	Multistage testing conducted by developers, evaluators and accessibility specialists; integrates model testing, accessibility evaluation, field testing and usability assessment	Testing and monitoring throughout the system life cycle	D—domains and testing stages are explicitly described and supported by an extensive conceptual and methodological basis	ND—the complete framework has not yet been empirically evaluated as an integrated procedure	ND	ND
2026	SHARA-WoZ	Multistakeholder procedural framework	Wizard of Oz procedure comparing manual, semi-autonomous and autonomous robot operation; the initial application involved experts rather than direct participation by older intended users	Prototyping and early comparison of alternative autonomy configurations	PD—structured development and expert involvement documented, but direct intended-user contribution remains limited	PD—initial laboratory application documented	ND	PD—laboratory implementation documented; real-world feasibility remains unestablished
2025	AI-enabled AT Framework	Principle-based framework	Participatory framework intended to guide stakeholders involved in the design, selection, implementation and	Across design, selection, implementation and review	D—content relevance supported by a participatory development process and	ND—empirical application of the complete framework	ND	ND

Year	Approach	Type	Persons involved and operational format	Primary stage or timing	Development transparency and content relevance	Empirical application of the complete approach	Procedural reproducibility	Feasibility
			review of *AI-enabled assistive technologies*		clearly articulated domains	has not been documented		
2025	Value Preference Profiles and Ethical Compliance Quantification	Ethics-by-design and simulation procedure	Construction of individual value-preference profiles and simulation-based comparison of prospective automated actions for persons with dementia	Prospective design and evaluation before implementation of automated actions	D—procedure derived from empirical preference data and explicitly described analytical stages	PD—profiles and simulations were applied, but external evaluation remains limited	PD—coding and protocol-verification procedures provide partial evidence	PD—simulation use documented; routine and real-world application remain unestablished
2025	X-HAZOP	Participatory structured procedure	Stakeholder workshops and structured analysis of deviations, ethical risks and social consequences in assistive-robot scenarios	Prospective risk analysis during design and development	PD—domains and participatory stages are described, with proof-of-concept support	PD—proof-of-concept applications documented	ND—consistency across groups and evaluators has not been established	PD—workshop-based implementation documented
2021	AUSUS Evaluation Framework	Multidimensional framework	Framework-based assessment of accessibility, usability, social implications and user experience in socially assistive robotics	Design, experimentation and field evaluation	PD—domains are articulated, but evidence supporting their completeness remains limited	PD—application to the CLARC robot and a subsequent real-world evaluation involving two socially assistive robots documented; breadth of application	ND	PD—applications in experimental and real-world settings documented, but limited to a small number of robotic systems and service contexts

Year	Approach	Type	Persons involved and operational format	Primary stage or timing	Development transparency and content relevance	Empirical application of the complete approach	Procedural reproducibility	Feasibility
						remains limited		
2020	Acceptance Test for Assistive Robots	Structured observational procedure	Observation of behavioural and affective responses of persons with dementia during interaction with a socially assistive robot	During and immediately after a brief interaction	PD—structured observation criteria are described, but formal development evidence remains limited	PD—the complete procedure was applied in a brief controlled trial	ND—formal inter-observer agreement has not been documented	D—feasibility demonstrated in a controlled experimental setting

Table S3. Target populations, technologies, AI components and properties evaluated across the included evaluation approaches[4].

Panel A. Target populations, technologies and basis for inclusion.

Year	Approach	Target population	Target technology or application field	Principal assistive functions addressed	Basis for inclusion in the AI-enabled assistive technology corpus
2026	AI TEVV for Accessibility Framework	Persons with disabilities and users with accessibility requirements; not restricted to a specific diagnostic condition	Assistive and general-purpose AI systems undergoing accessibility testing and evaluation	Access to information and communication, learning, mobility and other accessibility-related functions, depending on the system evaluated	Developed specifically for testing, evaluating, verifying and validating the accessibility of AI systems, including AI-enabled assistive applications
2026	SHARA-WoZ	Older adults as intended users; the initial evaluation involved expert stakeholders rather than direct participation by the intended users	Socially assistive robots evaluated through simulated manual, semi-autonomous and autonomous modes	Assistance with everyday activities, social interaction, companionship and support in care-related contexts	Developed to prototype and compare prospective automated functions and levels of autonomy in socially assistive robots through Wizard of Oz methods
2025	AI-enabled AT Framework	Persons with disabilities across application domains; not restricted to a specific diagnostic condition	*AI-enabled assistive technologies* incorporating speech, language, vision, classification, prediction, generation, planning or adaptive functions	Communication, access to information, mobility, manipulation, learning, monitoring and support for everyday activities	Developed specifically to guide the design, selection, implementation and review of *AI-enabled assistive technologies*
2025	Value Preference Profiles and Ethical Compliance Quantification	Persons with dementia	AI-based monitoring and assistive technologies intended for domestic and care contexts	Support for everyday activities, monitoring, safety and care-related decision making	Developed specifically to compare prospective automated actions with individual value-preference profiles in AI-based assistive systems for dementia care

[4] The target population refers to the persons for whom the evaluation approach or the evaluated technology was intended and does not necessarily correspond to the participants involved in the development or validation studies. For instruments originally developed within the broader assistive technology field, the table reports the specific robotic, intelligent or AI-enabled applications used to establish their relevance to the review. The presence of robotic or automated functions was not considered equivalent to the explicit evaluation of artificial intelligence. "No AI-specific property is evaluated separately" indicates that the algorithmic or automated component may be present in the technology but is assessed only through the general properties, interaction or outcomes of the system as a whole. Protocolled use indicates a planned application and was not treated as evidence of a completed empirical evaluation.

Year	Approach	Target population	Target technology or application field	Principal assistive functions addressed	Basis for inclusion in the AI-enabled assistive technology corpus
2025	X-HAZOP	Persons with disabilities and stakeholders involved in domestic, healthcare and care scenarios; not restricted to a specific diagnostic condition	Assistive robots involving autonomous or automated behaviour	Mobility, manipulation, monitoring, safety and assistance in domestic or care-related activities	Developed specifically for participatory prospective analysis of ethical hazards associated with autonomous functions and behaviour in assistive robots
2022	Robot Era Inventory	Older adults	Socially assistive robots, initially evaluated in relation to the Robot-Era platform	Social interaction, companionship, monitoring, service provision and support for everyday activities	Developed specifically to assess older adults' acceptance and experience of socially assistive robots. The instrument evaluates the robotic system as a whole rather than isolating its AI component
2021	AUSUS Evaluation Framework	Older adults, service users and stakeholders involved in healthcare and care settings	Socially assistive robots, including the CLARC robot and subsequent applications involving robots integrated into a retirement-home context	Interaction with services, guidance, information provision, support in care settings and accessible use of robotic assistance	Developed and applied specifically within socially assistive robotics, including experimental and real-world service contexts
2021	UNRAQ	Older adults and stakeholders involved in services supporting older adults, including relatives and professionals	Social and assistive robots intended for use in older-person care	Identification of needs, requirements and expectations concerning assistance, monitoring, safety, companionship and everyday support	Developed specifically for the multistakeholder assessment of needs, requirements, abilities and expectations relating to social robots in older-person care
2020	Acceptance Test for Assistive Robots	Persons with dementia	Socially assistive robots used during structured therapeutic interactions	Social interaction, cognitive stimulation, companionship and support during structured activities	Developed and empirically applied to determine the suitability and acceptance of assistive-robot functions during interactions with persons with dementia
2017	QUEAD	Users of physical assistive devices and robotic manipulators; not restricted to a specific diagnostic condition	Physical assistive devices, robotic manipulators and alternative device-control configurations	Manipulation, task execution, physical assistance and control of assistive devices	Developed specifically for physical human–robot interaction and the evaluation of robotic assistive devices. It was retained under the eligibility criterion covering assistive robotics; the original validation studies did not evaluate an AI component separately

Year	Approach	Target population	Target technology or application field	Principal assistive functions addressed	Basis for inclusion in the AI-enabled assistive technology corpus
2016	PYTHEIA	Users of rehabilitation and assistive robots or devices; not restricted to a specific diagnostic condition	Rehabilitation robots, robotic rollators and other assistive robotic devices, including systems providing intelligent navigation assistance	Rehabilitation, mobility, navigation, manipulation and task support	Developed specifically for rehabilitation and assistive robotics and subsequently applied to robotic rollators incorporating cognitive and intelligent navigation assistance
2010	Almere Model	Older adults	Socially assistive robots, screen agents and intelligent social agents	Social interaction, companionship, information provision, monitoring and everyday assistance	Developed specifically to assess acceptance of assistive social-agent technologies by older adults and subsequently applied across socially assistive robotic and intelligent-agent contexts
2002	IPPA	Assistive technology users; not restricted to a specific diagnostic condition	General assistive technologies, including robot manipulators and socially assistive or therapeutic robots	Individually prioritised activities and everyday problems, depending on the technology provided	Included through documented applications to robotic assistive systems, including robot manipulators and robots used to support individual therapeutic or educational goals. IPPA itself remains technology-agnostic and does not require an AI component
2002	PIADS	Assistive technology users; not restricted to a specific diagnostic condition	General assistive technologies, including robotic assistive devices and planned applications involving AI-enabled robotic systems	Mobility, communication, access to information and other functions, depending on the technology provided	Included through documented robotic assistive-technology applications and protocolled use in research involving AI-enabled robotic technology. Completed empirical evidence specific to *AI-enabled systems* remains limited
2002	QUEST 2.0	Assistive technology users; not restricted to a specific diagnostic condition	General assistive technologies, including robotic mobility systems and intelligent robotic rollators	Functions vary according to the device; the instrument assesses satisfaction with the technology and associated service provision	Included through documented application to robotic assistive systems, including the MOBOT rollator incorporating cognitive assistance and multimodal human–robot interaction
2001	ATD PA	Persons with disabilities and assistive technology users; not restricted to a specific diagnostic condition	General assistive technologies, including robotic mobility systems and protocolled AI-enabled robotic applications	Needs assessment, person–technology matching and support for meaningful activities across application domains	Included through documented application to the MOBOT robotic mobility assistant and protocolled use in research involving AI-enabled robotic assistive technology. The instrument itself is technology-agnostic

Panel B. AI or automated components and evaluated properties.

Year	Approach	AI or automated component incorporated into the technology	General technology or interaction properties evaluated	AI-specific properties evaluated
2026	AI TEVV for Accessibility Framework	Model-based speech, language, vision, classification, prediction, generation and adaptive functions, depending on the system evaluated	Accessibility, usability, technical performance, safety and performance under field-testing conditions	Output quality, accuracy, robustness, bias, failure modes and variation in performance across users, groups, tasks and contexts are translated into explicit testing stages
2026	SHARA-WoZ	Prospective automated robot control simulated through manual, semi-autonomous and autonomous operating modes; implementation of an operational AI model is not required during the Wizard of Oz evaluation	Interaction quality, stakeholder judgements, task execution, integration features and consequences of different control configurations	Levels of autonomy and operating modes are explicitly compared; accuracy, robustness, bias and other properties of an implemented AI model are not independently tested
2025	AI-enabled AT Framework	Speech, language, vision, classification, prediction, generation, planning and adaptive functions across assistive-technology domains	Accessibility, usefulness, safety, privacy, value, rights, choice, control and overall quality	Quality and reliability of AI functions, safety, equity, transparency, privacy and allocation of choice and control are explicitly addressed at the level of framework principles
2025	Value Preference Profiles and Ethical Compliance Quantification	AI-based automated planning and decision making concerning prospective monitoring or assistive actions	Contextual appropriateness, ethical consequences and consistency with the person's preferences	Alignment between prospective automated actions and the individual value-preference profile is explicitly evaluated
2025	X-HAZOP	Autonomous perception, navigation, action selection or decision making, depending on the assistive-robot scenario	Safety, ethical and social risks, stakeholder consequences and deviations from intended operation	Risks associated with autonomy and automated behaviour are explicitly examined; model accuracy, robustness and bias are not independently tested
2022	Robot Era Inventory	Interactive, sensing, navigation or adaptive robot functions incorporated into the Robot-Era system	Acceptance, perceived usefulness, trust, attitudes and experience of interaction	No AI-specific property is evaluated separately from the socially assistive robot as a whole
2021	AUSUS Evaluation Framework	Autonomous navigation, interaction and service-related robot functions in the documented applications	Accessibility, usability, social acceptance, user experience, societal impact and field performance	No AI-specific property is evaluated separately from the complete robotic system
2021	UNRAQ	Social-interaction, monitoring, navigation or adaptive functions, depending on the robot considered; the questionnaire does not prescribe a specific AI architecture	Needs, requirements, abilities, expectations, usefulness, acceptability and organisational or contextual factors	No AI-specific property is evaluated separately from the social or assistive robot as a whole

Year	Approach	AI or automated component incorporated into the technology	General technology or interaction properties evaluated	AI-specific properties evaluated
2020	Acceptance Test for Assistive Robots	Automated or pre-programmed robot behaviour and individual robot functions during structured therapeutic interaction	Observable behavioural and affective responses, rejection, acceptance and suitability of the robot and its functions	No AI-specific property is evaluated separately from robot behaviour and interaction
2017	QUEAD	Robotic actuation and alternative device-control modes in physical human–robot interaction; an AI component was not specified or isolated in the original validation studies	Usability, comfort, physical effort, effectiveness, control and quality of task interaction	No AI-specific property is evaluated separately from device performance, control and interaction
2016	PYTHEIA	Robotic assistance and automated control; documented applications include intelligent navigation, cognitive assistance and sensor-supported interaction	Usability, satisfaction, perceived usefulness and overall experience of the rehabilitation or assistive system	No AI-specific property is evaluated separately from the overall performance and user experience of the robotic device
2010	Almere Model	Interactive, autonomous or adaptive functions incorporated into socially assistive robots and intelligent social agents	Perceived usefulness, perceived ease of use, enjoyment, trust, social presence, attitudes and intention to use	No AI-specific property is evaluated separately from overall acceptance of the system
2002	IPPA	The instrument is technology-agnostic; documented applications include robot manipulators and therapeutic or socially assistive robot functions	Change in the difficulty of individually prioritised activity problems	No AI-specific property is evaluated separately. Outcomes are attributed to the assistive intervention as a whole
2002	PIADS	The instrument is technology-agnostic; included applications involve robotic assistance, while an AI-enabled robotic application is represented principally by a study protocol	Psychosocial impact through competence, adaptability and self-esteem	No AI-specific property is evaluated separately from the psychosocial effects attributed to the assistive device as a whole
2002	QUEST 2.0	The instrument is technology-agnostic; the documented MOBOT application involved robotic mobility, cognitive assistance, multimodal sensing and adaptive human–robot interaction	Satisfaction with the device and service provision, including effectiveness, ease of use, comfort and perceived reliability	No AI-specific property is evaluated separately from satisfaction with the complete robotic or assistive system.
2001	ATD PA	The instrument is technology-agnostic; documented applications involve robotic mobility and cognitive assistance, while further AI-enabled robotic use is represented partly by protocolled research	Person–technology matching, personal characteristics, activities, environmental support and factors associated with use or abandonment	No AI-specific property is evaluated separately from the quality of person–technology matching and the assistive system as a whole
